# Anthelmintic Activity, Cytotoxicity, and Phytochemical Screening of Plants Used to Treat Digestive Parasitosis of Small Ruminants in Benin (West Africa)

**DOI:** 10.3390/ani12192718

**Published:** 2022-10-10

**Authors:** Esaïe Tchetan, Pascal Abiodoun Olounladé, Erick Virgile Bertrand Azando, Hafiz Abdul Khaliq, Sergio Ortiz, Alban Houngbeme, Géorcelin Goué Alowanou, Bossima Ivan Koura, Guénolé Coovi Akouedegni, Marcel Romuald Benjamin Houinato, Sylvie Mawule Hounzangbe-Adote, Fernand Ahokanou Gbaguidi, Joëlle Quetin-Leclercq

**Affiliations:** 1 Laboratoire d’Ethnopharmacologie et de Santé Animale, Faculté des Sciences Agronomiques, Université d’Abomey-Calavi, Cotonou 01 BP 526, Benin; 2 Laboratoire de Biotechnologie et d’Amélioration Animale, Faculté des Sciences Agronomiques, Institut des Sciences Biomédicales Appliquées (ISBA), Université d’Abomey-Calavi, Cotonou 01 BP 526, Benin; 3 Laboratoire de Chimie Organique et Chimie Pharmaceutique, UFR Pharmacie, Faculté des Sciences de la Santé, Université d’Abomey-Calavi, Cotonou 01 BP 188, Benin; 4 Pharmacognosy Research Group, Louvain Drug Research Institute, Université catholique de Louvain (UCLouvain), Avenue E. Mounier, B1.72.03, B-1200 Brussels, Belgium; 5 Unité de Recherche en Zootechnie et Système d’Elevage (URZoSE), Laboratoire des Sciences Animale et Halieutique (LaSAH), Ecole de Gestion et d’Exploitation des Systèmes d’Elevage (EGESE), Université Nationale d’Agriculture (UNA), Porto-Novo 01 BP 55, Benin; 6 Laboratoire d’Écologie, de Santé et de Productions Animales, Département des Sciences et Techniques de Production Animale et Halieutique, Faculté d’Agronomie, Université de Parakou, Cotonou 01 BP 2115, Benin; 7 Laboratoire des Recherches Pluridisciplinaires de l’Enseignement Technique, Ecole Normale Supérieure de l’Enseignement Technique, Université Nationale des Sciences, Technologies, Ingénierie et Mathématiques, Abomey BP 2282, Benin; 8 Ecole de Gestion et d’Exploitation des Systèmes d’Elevage, Université Nationale d’Agriculture, Kétou BP 43, Benin; 9 Ecole des Sciences et Techniques de Production Animale, Faculté des Sciences Agronomiques, Université d’Abomey-Calavi, Abomey-Calavi 01 BP 526, Benin

**Keywords:** anthelmintic activity, medicinal plants, *Haemonchus contortus*, cytotoxicity, secondary metabolites

## Abstract

**Simple Summary:**

In the present study, we explored the anthelmintic activity, cytotoxicity, and chemical composition of the main plants used by small ruminant breeders in Benin to treat digestive parasitosis. The results obtained are relevant in the sense that they will allow a rational use of the plants studied in the control of digestive parasites but also to initiate the process of identification of new anthelmintic molecules in the context of the development of resistance against the most currently used anthelmintic molecules.

**Abstract:**

Medicinal plants continue to be used alone or in combination with veterinary drugs to treat animal ailments, especially in developing countries where livestock farmers often lack access to modern veterinary services and drugs. In addition, digestive parasitosis remain a major constraint for small ruminant livestock. The objective of this study was to screen the anthelmintic activity of the main plants used in the treatment of the digestive parasitosis of small ruminants in Benin. A total of 40 extracts were prepared using the successive maceration of 10 plants in four solvents of increasing polarity. The phytochemical screening of the plants was performed, and the anthelmintic activity of the extracts was evaluated on L3 larvae of *Haemonchus contortus*. The cytotoxicity of the 40 extracts was determined on WI38 noncancerous fibroblast cells using the MTT assay, and the total phenol content (TPC), total flavonoid content (TFC), and condensed tannin content (CTC) were quantified in the most effective extracts using colorimetric methods. The results show that the plants contained tannins, flavonoids, and triterpenoids which may, in part, justify their anthelmintic activities. All plants gave active extracts at the highest concentration tested (1200 µg/mL). Methanol (MeOH) extracts were, in general, more effective than the hexane (HEX), dichloromethane (DCM), and aqueous (H_2_O) ones in inhibiting larval migration, with the MeOH extracts of *Terminalia leiocarpa*, *Adansonia digitata*, and *Momordica charantia* being the most effective. Nevertheless, the MeOH extract of *M. charantia* was highly cytotoxic at the concentration of 100 µg/mL. The anthelmintic activity of *M. charantia*, *Vitex doniana*, and *Caesalpinia bonduc* was studied on *H. contortus* for the first time. These results provide scientific information that can be used for better valorization of the anthelmintic potential of the studied plants and to initiate the process of the identification of new anthelmintic molecules.

## 1. Introduction

Digestive parasitosis represent a major constraint for the breeding of ruminants, especially those fed on pasture [1]. Diseases caused by helminths are encountered in nearly 84% of small ruminant farms in southern Benin [2]. Pathologies due to gastrointestinal parasites evolve in a chronic manner, and clinical signs are only perceptible in the presence of a high parasite load. The symptoms of digestive parasitosis can range from digestive disorders (diarrhea, indigestion, and loss of appetite) to reduced productivity [3]. The artificial infestation of goats with an average of 5000 *Haemonchus contortus* larvae per animal resulted in decreased milk production of between 2.5 and 10% [3,4].

The control of digestive parasitosis relies mainly on chemotherapy in developed countries. However, the inaccessibility of veterinary drugs and services and the low purchasing power of livestock farmers in developing countries lead them to resort to ethnoveterinary medicine to treat animal diseases [5]. Ethnoveterinary surveys have been carried out to inventory the plants used to treat animal diseases [2,6,7]. Cross-checking the results of the ethnoveterinary surveys allowed us to identify the ten main plants used by farmers to treat digestive diseases of small ruminants in Benin. These are *Terminalia leiocarpa* (DC.) Baill., *Momordica charantia* L., *Caesalpinia bonduc* (L.) Roxb., *Adansonia digitata* L., *Azadirachta indica* A. Juss., *Crossopteryx febrifuga* (Afzel. ex G. Don) Benth., *Zanthoxylum zanthoxyloïdes* (Lam.) Zepern. & Timler, *Morinda lucida* Benth., *Vitex doniana* Sweet, and *Spondias mombin* L. It is therefore necessary to build up a solid scientific database of these plants for their better use in the treatment of ruminant digestive parasitosis.

The validation of remedies used in traditional medicine involves the verification of presumed biological activities, phytochemical analysis, and toxicity assessment [5,8]. The anthelmintic activity of *T. leiocarpa*, *A. indica*, *S. mombin*, and *Z. zanthoxyloïdes* has been previously evaluated [9,10,11,12,13,14,15]. However, the anthelmintic activity of *A. digitata*, *M. charantia*, *C. bonduc*, *C. febrifuga*, *M. lucida*, and *V. doniana* has not been sufficiently documented. It is therefore important to evaluate and compare the anthelmintic activity of these plants in order to better valorize them in ethnoveterinary medicine. Furthermore, the evaluation of the anthelmintic activity of the other plants is also relevant, especially as the presence and concentrations of secondary plant metabolites responsible for biological activity may vary according to the environment [16].

The screening of these selected plants will allow the validation of their traditional uses and initiate the process of identifying new anthelmintic molecules to replace chemical drugs in the face of resistance development [17]. In addition, the extension of the validated remedies in the treatment of digestive parasitosis will reduce the pressure on the previously validated plants and limit the development of resistance, as remedies can be used alternately and often contain several active molecules with different targets [9].

Some medicinal plants produce toxic secondary metabolites that may have adverse effects. Thus, it is desirable to assess the toxicity of traditional herbal extracts or remedies when screening for biological activity in order to determine the adverse effects as well as the lethal dose [18]. The cytotoxicity of traditional extracts or remedies can be assessed on different cell types [19,20,21], but WI38 cells (human noncancerous fibroblast cell lines) remain one of the oldest and most used [22,23].

The main objective of the present study was to screen the anthelmintic activity of the most commonly used plants to treat the digestive parasitosis of small ruminants in Benin. Specifically, the aims were to: (i) identify the main classes of secondary metabolites present in the selected plants, (ii) evaluate the anthelmintic activities of these plant extracts on *H. contortus* larvae, (iii) determine their cytotoxicity on WI38 cell lines.

## 2. Materials and Methods

### 2.1. Plant Identification and Collection

**Figure 1:**
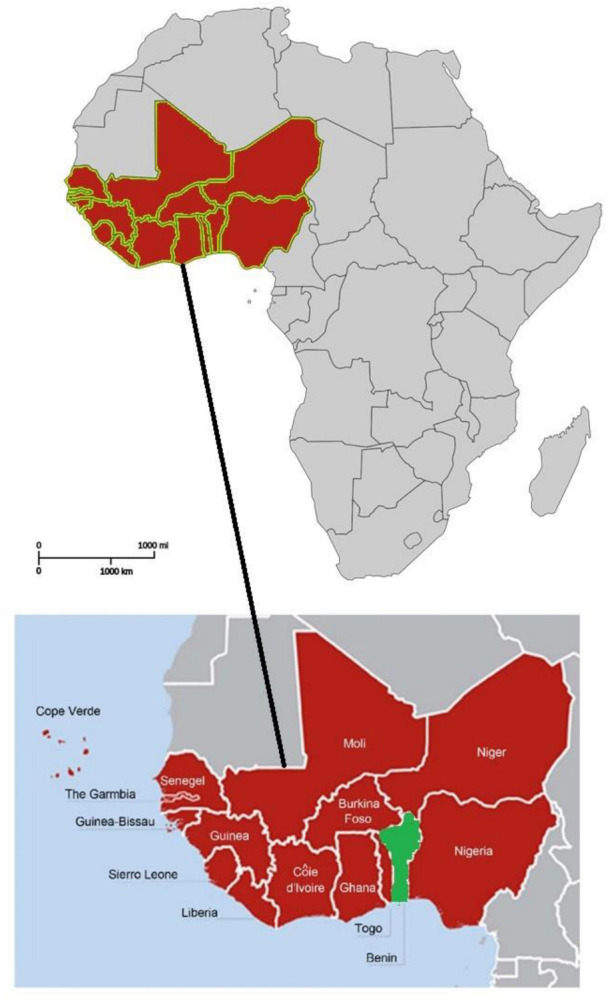
Map showing the geographical location of Benin in West Africa. Source: adapted from [24,25].

Fresh leaves of *T. leiocarpa*, *M. charantia*, *C. bonduc*, *A. digitata*, *A. indica*, *C. febrifuga*, *Z. zanthoxyloïdes*, *M. lucida*, *V. doniana*, and *S. mombin* were collected in their natural habitats. Those of *T. leiocarpa*, *C. febrifuga*, *A. digitata*, and *V. doniana* were collected from the municipality of N’Dali (Northern Benin). Those of *A. indica*, *Z. zanthoxyloïdes*, *M. lucida*, *C. bonduc*, *M. charantia*, and *S. mombin* were collected from the municipality of Bopa (Southern Benin). Samples were collected in April 2019. Benin is located in West Africa at latitude 9.30769 and longitude 2.315834 (Figure 1). The collected samples were authenticated at the National Herbarium of Benin (NHB), University of Abomey-Calavi, Benin (Table 1). Herbarium specimens were kept at NHB. The leaves were washed with water, dried in the laboratory at 25 °C for 15 days, and ground in a mill with a 0.5 mm diameter mesh. The resulting powders were stored at 25 °C in hermetically sealed boxes until use.

### 2.2. Phytochemical Screening of Plants

Phytochemical screening was carried out using differential reactions (staining and precipitation) of the main groups of chemical compounds contained in the plants according to the classical methods described by Houghton and Raman [26]. The samples were analyzed for the presence of different classes of secondary metabolites including flavonoids, alkaloids, tannins, anthocyanins, saponosides, triterpenes, and anthraquinones. The methodology used has been previously described in the literature [27,28].

### 2.3. Extraction Procedure

The extraction was carried out using successive macerations with HEX, DCM, MeOH, and H_2_O (solvents with increasing polarity). Organic solvents (HPLC grade) were purchased from VWR International (Radnor, PA, USA). In summary, 250 mL of HEX was added to 50 g powder of each sample, and the mixture was macerated on a shaker for 12 h. After filtration, a second portion of 250 mL HEX was used for a further 12 h maceration under shaking. The same procedure was repeated for each solvent on the same sample. The extracts obtained using organic solvents were evaporated using a rotary evaporator, while the H_2_O extracts were freeze-dried. The obtained extracts were weighed, transferred to sample bottles, labelled, and stored at 4 °C until use. To perform the tests, they were solubilized in dimethylsulfoxide (DMSO) at 20 mg/mL.

### 2.4. Screening for Anthelmintic Activity of Extracts

Anthelminthic potency of obtained extracts was evaluated on live *H. contortus* larvae.

#### 2.4.1. Obtaining Infective Larvae

Infective larvae (L3) of *H. contortus* were obtained using the technique described in the literature [12,29]. Briefly, droppings from a sheep artificially infested with a pure strain of *H. contortus* (obtained from the Laboratoire d’Ethnopharmacologie et de Santé Animale, University of Abomey-Calavi) were cultured at room temperature in the laboratory for ten days. The Baermann device was set up to extract L3 larvae from the cultured droppings. The resulting larvae were stored in a refrigerator at 4 °C for 3 months before use.

#### 2.4.2. Larval Migration Inhibition Assay

**Figure 2:**
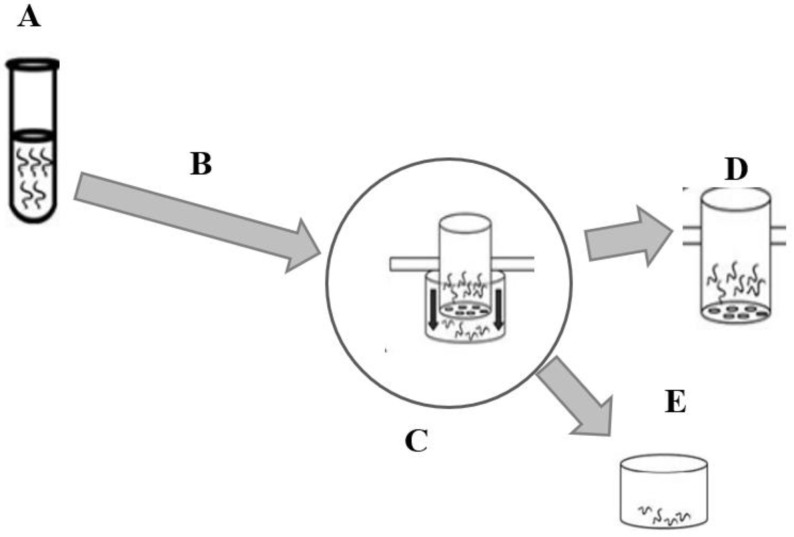
Larval migration inhibition assay (LMIA) illustration. (**A**) Incubation of infective larvae (L3) with controls and extracts at different concentrations for 3 h (25 °C). (**B**) Successive washing (3 times) of incubated larvae by centrifugation (67× *g*) with phosphate buffered saline (PBS). (**C**) Transfer of washed larvae to inserts (20 µm diameter) for migration. (**D**) After 3 h, the larvae in the inserts were discarded. (**E**) Larvae count in the conical tube at the bottom of the inserts. Source: adapted from Zarate Rendon [32].

The screening of the anthelmintic activity of the extracts was performed using the larval migration inhibition assay (LMIA) (Figure 2) as described in the literature [30,31]. Larvae (1000 L3/mL) were incubated with the extracts at different concentrations (150, 300, 600, and 1200 µg/mL) in phosphate buffered saline (PBS) solution. Dimethylsulfoxide (1%) and levamisole (250 µg/mL) were used as negative and positive controls, respectively. After 3 h of incubation at 25 °C, the larvae were washed three times successively by centrifugation (67× *g*) with PBS (pH 7.2) and placed on inserts (20 µm diameter) for migration for 3 h. The inserts were previously deposited in canonical tubes containing the PBS solution. After 3 h, the larvae contained in the inserts were discarded, and those that migrated into the canonical tubes below the inserts were recovered and counted under the microscope (Figure 1). The test was conducted in four replicates for each concentration of extract and controls and repeated three times. The rate of inhibition of larval migration was calculated according to the following formula:

(1) $$A=\frac{T-M}{T}\times 100$$

where A is the rate of inhibition of larval migration, T is the total number of larvae deposited on the insert, and M is the number of larvae counted in the canonical tube.

### 2.5. Cytotoxicity Evaluation of Crude Extracts

The cytotoxicity of crude extracts was evaluated on WI38 cells (human noncancerous fibroblast cell lines) (obtained from LGC standards, Molsheim, France) following the methodology described by Stévigny et al. [33]. Solubilized extracts were diluted in culture medium to obtain tested concentration. Briefly, cells (5000 cells/well) were treated with the extracts at a concentration of 100 µg/mL at 37 °C in 96-well plates in DMEM (Dulbecco’s Modified Eagle Medium) supplemented with 10% inactivated fetal calf serum and 1% penicillin/streptomycin (purchased from Lonza, Verviers, Belgium). After 72 h of incubation, the medium was replaced by 100 µL of MTT [3-(4,5-dimethylthiazol-2-yl)-2,5-diphenyl tetrazolium bromide] tetrazolium salt (purchased form Sigma-Aldrich, Bornem, Belgium) to measure cellular metabolic activity, an indicator of cell viability [18]. MTT solution was prepared by dissolving 15 mg of MTT in 50 mL (5 mL of PBS and 45 mL of DMEM). After 45 min, MTT was removed from the wells, an equal volume of DMSO was added, and the absorbance was measured with a spectrophotometer (SpectraMax M3) at 570 and 620 nm to quantify the formazan formed by the reduction in MTT. Camptothecin and DMSO (0.5%) were used, respectively, as positive and negative controls. The test was repeated three times in duplicate.

### 2.6. Quantification of Secondary Metabolites

#### 2.6.1. Total Phenol Content (TPC)

The determination of TPC was performed by the colorimetric method using the Folin–Ciocalteu reagent [34]. Briefly, samples were solubilized in DMSO (MeOH and DCM extracts), ethanol (EtOH) (HEX extracts), or H_2_O (H_2_O extracts) at concentrations of 4, 1, and 0.5 mg/mL. Gallic acid was prepared at different concentrations in DMSO (1 to 0.0015 mg/mL) and used for the calibration curve. In each well, 25 µL of sample, gallic acid, or the blanks (DMSO, EtOH, or H_2_0) were added, plus 125 µL of Folin–Ciocalteu reagent (previously diluted in water, 10% *v*/*v*) and 100 µL of sodium bicarbonate (7.5% *w*/*v* in distilled water). For blanks (control wells), the Folin–Ciocalteu reagent was replaced by 125 µL of distilled water. The plates were protected from light with aluminium foil and incubated at room temperature for 30 min. The plates were read by spectrophotometer (SpectraMax M3) at 765 nm. The test was repeated three times. The concentration of TPC was expressed as mg of gallic acid equivalent per g of extract.

#### 2.6.2. Total Flavonoid Content (TFC)

Total flavonoid content (TFC) was quantified by the colorimetric method using aluminium chloride reagent. The protocol used is based on that described by Arora and Itankar [35] with a slight modification. Briefly, the extracts were solubilized in DMSO (MeOH and DCM extracts), EtOH (HEX extracts), or H_2_O (H_2_O extracts) at concentrations of 4, 1, and 0.5 mg/mL. Fifty microliters of sample were placed in each well, plus 160 µL of EtOH, 20 µL of aluminium chloride (1.8% *w*/*v* in distilled water), and 20 µL of sodium acetate (820.3 mg in 100 mL of distilled water). Sodium chloride and sodium acetate were replaced by 40 µL of deionized water in the blanks (control wells). Plates were incubated at room temperature for 40 min and read by spectrophotometer (SpectraMax M3) at 415 nm. Quercetin was used as standard (initial concentration: 1 to 0.0015 mg/mL in DMSO) to obtain the calibration curve. The test was repeated three times. Total flavonoid content was expressed as mg quercetin equivalent per g extract.

#### 2.6.3. Condensed Tannin Content (CTC)

The determination of CTC was carried out according to the methodology described by Feliciano et al. [36]. Briefly, the extracts were solubilized in DMSO (MeOH and DCM extracts), EtOH (HEX extracts), or H_2_O (H_2_O extracts) at concentrations of 4, 1, and 0.5 mg/mL. Catechin was prepared at different concentrations in MeOH (initial concentrations: 15.3 to 2.6 µg/mL) as standard and used to obtain the calibration curve. DMAC reagent [4-(dimethylamino)cinnamaldehyde] was prepared (1 mg/mL in acidified EtOH) and used extemporaneously [36]. Seventy microliters of sample, blanks (DMSO, EtOH, or H_2_0) and the standard (at different concentrations) were deposited in each well plus 210 µL of DMAC reagent. The DMAC reagent was replaced by EtOH in the control wells. The plates were protected from light and incubated at room temperature for 8 min. The reading was taken with a spectrophotometer (SpectraMax M3) at 640 nm. The concentration of CTC was expressed as mg catechin equivalent per g of extract. The test was repeated three times.

### 2.7. Statistical Analysis

The mean rate of inhibition of larval migration was calculated per concentration of each extract tested and then per control. The mean for each dose tested was compared to the negative control (DMSO). We checked the normality and equality of the variance in order to perform an analysis of variance (ANOVA). As these conditions were not fulfilled even after transformation of the data, we used Mann–Whitney test for the comparison of the medians. Analyses were performed in R software [37], and differences were considered significant at the 5% level. For cytotoxicity, data were presented as means ± standard deviations for each extract. These methods were described by Olounladé et al. [13].

## 3. Results

### 3.1. Secondary Metabolites of the Studied Plants

Table 2 presents the main classes of the secondary metabolites detected in the leaf powders of *T. leiocarpa*, *M. charantia*, *C. bonduc*, *A. digitata*, *A. indica*, *C. febrifuga*, *Z. zanthoxyloïdes*, *M. lucida*, *V. doniana*, and *S. mombin*. All plants studied contained tannins and reducing compounds. On the other hand, flavonoids were detected in all leaf powders except those of *A. digitata* and *Z. zanthoxyloïdes*. Alkaloids were present in the leaf powders of *C. bonduc*, *V. doniana*, *Z. zanthoxyloïdes*, and *S. mombin*.

### 3.2. Extraction Yield

A total of 40 extracts were prepared. Table 3 shows the yield of the extractions according to the plants and solvents. Extraction yields increased with increasing solvent polarity for the samples of *Z. zanthoxyloïdes*, *V. doniana*, *S. mombin*, *M. lucida*, *M. charantia*, and *C. febrifuga*. The yields of DCM extracts were higher than those of other solvents for the *T. leiocarpa* and *A. digitata* samples. On the other hand, the yields of the H_2_O extracts of *C. bonduc* and *A. indica* were higher than those obtained from the same plants with the other solvents (Table 3).

### 3.3. Anthelmintic Activity of the Extracts

#### 3.3.1. Effect of the HEX Extracts on the Migration of *H. contortus* Larvae

**Figure 3:**
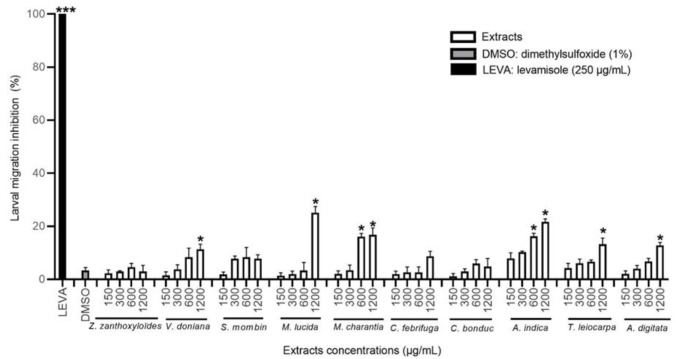
Migration inhibition rate (in %) of *H. contortus* larvae treated with hexane extracts at different concentrations, DMSO, and levamisole. Asterisks indicate a significant difference compared to the negative control (DMSO) (* *p* < 0.05, ** *p* < 0.01, *** *p* < 0.001). Each histogram represents the mean ± standard deviation from 3 × 3 values.

The HEX extracts of *A. indica*, *M. charantia*, *M. lucida*, *T. leiocarpa*, *A. digitata*, and *V. doniana* significantly inhibited the migration of *H. contortus* L3 larvae compared to the negative control (*p* < 0.05). This inhibitory effect was dose-dependent at the concentrations tested, but the activity did not exceed 30% inhibition (Figure 3 and Table 4). The *A. indica* extract was the most effective with *M. charantia* from 600 µg/mL, while the *M. lucida* extract was the more active at the highest concentration tested (with about 25% inhibition).

#### 3.3.2. Effect of the DCM Extracts on the Migration of *H. contortus* Larvae

**Figure 4:**
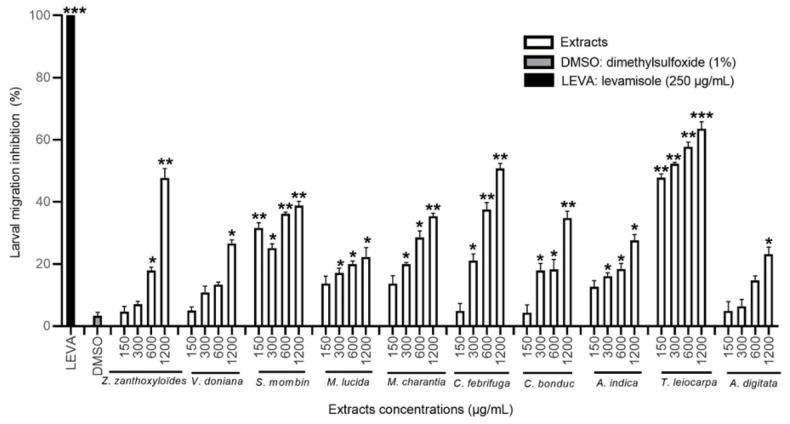
Migration inhibition rate (in %) of *H. contortus* larvae treated with dichloromethane extracts at different concentrations, DMSO, and levamisole. Asterisks indicate a significant difference compared to the negative control (DMSO) (* *p* < 0.05, ** *p* < 0.01, *** *p* < 0.001). Each histogram represents the mean ± standard deviation from 3 × 3 values.

All of the DCM extracts significantly inhibited the migration of *H. contortus* larvae (*p* < 0.05) compared to the negative control at the highest concentration at least. Extracts of *T. leiocarpa*, *S. mombin*, and *C. febrifuga* were the most effective (Figure 4), but the *Z. zanthoxyloïdes* extract activity at the highest concentration was also high. The DCM extract of *T. leiocarpa* was the most effective with almost 63% inhibition of *H. contortus* larval migration at the concentration of 1200 µg/mL (Table 4). The inhibitory effect of the different extracts was dose-dependent except for *S. mombin*.

#### 3.3.3. Effect of the MeOH Extracts on *H. contortus* Larval Migration

**Figure 5:**
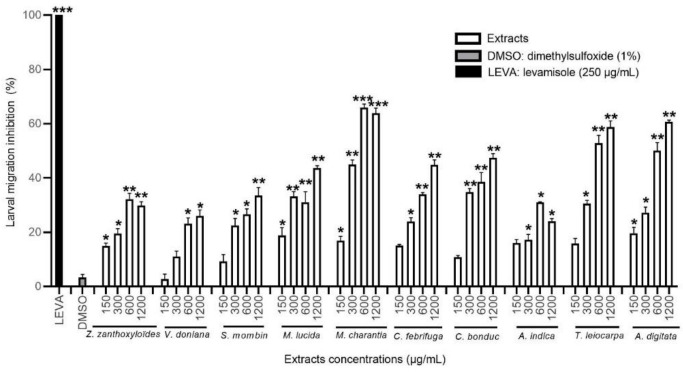
Migration inhibition rate (in %) of *H. contortus* larvae treated with methanol extracts at different concentrations, DMSO, and levamisole. Asterisks indicate a significant difference compared to the negative control (DMSO) (* *p* < 0.05, ** *p* < 0.01, *** *p* < 0.001). Each histogram represents the mean ± standard deviation from 3 × 3 values.

In a concentration-dependent manner, all of the ten MeOH extracts significantly reduced the migration of *H. contortus* L3 larvae compared to the negative control (*p* < 0.05). The extracts of *M. charantia*, *T. leiocarpa*, *C. bonduc*, *M. lucida*, and *A. digitata* were the most effective (Figure 5). The MeOH extract of *M. charantia* was one of the most active with a migration inhibition rate of 64% at a high concentration. The MeOH extracts of *T. leiocarpa* and *A. digitata* were very active with the respective inhibition rates of 58.7% and 60.6% (Table 4).

#### 3.3.4. Effect of the H_2_O Extracts on *H. contortus* Larval Migration

**Figure 6:**
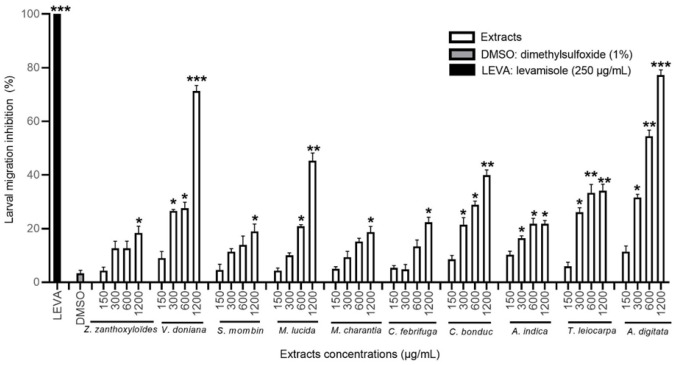
Migration inhibition rate (in %) of *H. contortus* larvae treated with aqueous extracts at different concentrations, DMSO, and levamisole. Asterisks indicate a significant difference compared to the negative control (DMSO) (* *p* < 0.05, ** *p* < 0.01, *** *p* < 0.001). Each histogram represents the mean ± standard deviation from 3 × 3 values.

All of the ten H_2_O extracts tested significantly reduced the migration of *H. contortus* larvae compared to the negative control (*p* < 0.05), and this inhibitory effect was dose-dependent. The extracts of *A. digitata* and *V. doniana* were the most effective, inhibiting more than 70% of the larval migration at the highest concentration tested (Figure 6). Similarly, the H_2_O extract of *M. lucida* was active on *H. contortus* larvae with an inhibition rate of 45.3% at the concentration of 1200 µg/mL (Table 4).

However, none of the extracts tested, even at the highest concentration, induced 100% inhibition similar to the positive control (levamisole).

### 3.4. Cytotoxicity of Extracts

**Figure 7:**
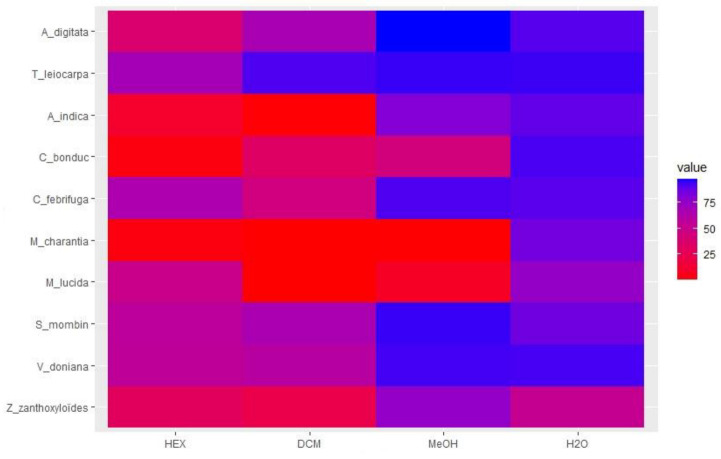
Heatmap of WI38 cells viability (compared to negative control considered as 100%) treated with extracts (100 µg/mL). HEX: hexane; DCM: dichloromethane; MeOH: methanol; and H_2_0: water. DMSO (0.5%) was used as a negative control.

The cytotoxicity results of the crude extracts at 100 µg/mL are presented in Figure 7. The HEX and DCM extracts of *Z. zanthoxyloïdes*, *M. lucida*, *M. charantia*, *C. bonduc*, and *A. indica* strongly reduced the viability of WI38 cells with less than 40% remaining viable (Figure 7). In contrast, the H_2_O extracts of all plants were considered not cytotoxic (>80% viability) to cells at the maximum concentration tested except for those of *Z. zanthoxyloïdes* with viability around 50%. *Terminalia leiocarpa*, *A. digitata*, *V. doniana*, *S. mombin*, and *C. febrifuga* were less cytotoxic irrespective of the type of extract, but the lipophilic extracts were often more cytotoxic than the more polar ones.

### 3.5. Determination of TPC, TFC, and CTC

Table 5 shows the concentrations of different samples in mg equivalent of gallic acid, quercetin, and catechin per gram of extract of all extracts of some selected plants. These results show that different extracts of *T. leiocarpa* were richer in phenolic compounds and flavonoids compared to *M. lucida* and *C. febrifuga* (Table 5).

## 4. Discussion

The present study investigated the anthelmintic potential of the most commonly used plants to control the digestive diseases of small ruminants in Benin in order to validate their traditional use and to initiate the process of identifying new anthelmintic compounds. This is particularly relevant in the context of resistance development against the most currently known anthelmintic molecules. The choice of plants studied was based on the results of ethnoveterinary surveys, and priority was given to the plants most cited by the farmers. This approach has a limitation in the fact that a plant may be very effective but not well known by farmers [6]. In addition, the screening of the anthelmintic activity of plants at the larval stage alone does not represent all parasite stages. Indeed, some plants can be active on larvae and others on eggs or adult worms. Levamisole, for example, is very effective on larvae and adult worms but ineffective on eggs [38]. *Haemonchus contortus* used in this study is the most pathogenic and predominant digestive parasite in small ruminant farms, especially in tropical countries. Therefore, it has been widely used as a model organism in the process of evaluating the anthelmintic activity of natural substances [12,29,31,39]. The concentrations used are those commonly tested in other publications [12,31].

The anthelmintic activity of 40 extracts from ten plants used in the treatment of the digestive parasitosis of small ruminants in Benin was evaluated. The results (summarized in Table 4) show that the DCM, MeOH, and H_2_O extracts significantly inhibited the migration of *H. contortus* larvae compared to the negative control, and this inhibitory effect was dose-dependent. The HEX extracts, in general, were less effective, and the inhibitory effect was only significant compared to the negative control at the highest (1200 µg/mL) concentration for certain plants. Similar results have been obtained in medicinal plants used in ethnoveterinary medicine in sub-Saharan Africa [17]. These results suggest that there are several compounds with anthelmintic properties of different polarity in each of the plants studied. Indeed, the phytochemical screening showed that they contain several classes of secondary metabolites, including tannins, alkaloids, flavonoids, and terpenes, which may account for a part of the observed biological activity. In general, the MeOH extracts were more effective in inhibiting larval migration. This can be justified by the high solubility of flavonoids and tannins in MeOH. In fact, tannins and flavonoids have been widely indexed in the anthelmintic activity of plants [40,41,42]. Results show that DCM, MeOH and H_2_O extracts of *T. leiocarpa*, having very interesting activity, are very rich in phenolic derivatives and flavonoids, which may account for at least a part of their activity. Nevertheless, Table 4 shows that the activity of extracts is not directly related to the phenolic derivatives/tannins/flavonoids contents. This indicates that different types of phenolic derivatives may have different activities and that polyphenols are not responsible for the total activity observed. Other compounds should be present and have an additive or synergistic effect and should be identified, in the search for new anthelmintic compounds.The most active extract at the lowest concentration was the DCM extract of *T. leiocarpa*. The MeOH extracts of *T. leiocarpa*, *M. charantia*, *M. lucida*, *A. digitata*, and *C. bonduc* were also very effective in inhibiting the migration of *H. contortus* larvae. Similarly, the H_2_O extracts of *V. doniana* and *A. digitata* were the most effective (Table 4).

The anthelmintic activity of certain plants studied has been widely evaluated. This is the case for *Z. zanthoxyloïdes*, whose anthelmintic activity has been extensively studied *in vitro* and *in vivo* on the digestive parasites of small ruminants (*H. contortus* and *Trichostrongylus colubriformis*) [12,13,43] and which was also found in our screening. In addition, the anthelmintic activity of *T. leiocarpa* has been widely explored [14,15,44,45]. Our results corroborate these data from the literature and suggest that the anthelmintic activity of this plant is due to the presence of tannins and flavonoids (Table 2) or other phenolic derivatives which may be present as polar compounds (i.e., heterosides) but also more lipophilic aglycone/esters forms given the high activity of the DCM extract. Ndjonka et al. [46] associated the anthelmintic activity of *T. leiocarpa* with the ellagic, gallic, and gentisic acids it contains. The results of some secondary metabolites quantification showed that different extracts of *T. leiocarpa* were rich in TPC, TFC, and CTC (Table 5). This could justify the effectiveness of the different extracts of this plant on the inhibition of larval migration. The data on the quantification of TFC and TPC in certain HEX and DCM extracts may be surprising, given the usual very low solubility of these types of compounds (often found bound to sugars) in these solvents, but some esters or lipophilic aglycone forms could be found in the DCM extracts. Another explanation may be related to the low specificity of the quantification methods used. Indeed, the Folin–Ciocalteu reagent interferes not only with phenolic compounds but also with several other reducing compounds, some of which are lipophilic, such as terpenic compounds [47,48]. Similarly, the aluminium chloride reagent used to quantify flavonoids is not specific to this class of compound. The reagent can interfere in an alkaline medium with any compound presenting carbonyl or hydroxyl groups [49].

Similar to *T. leiocarpa*, the anthelmintic activity of *S. mombin* has been widely described in the literature [10,50]. The results of our study confirm the anthelmintic property recognized in this plant and suggest that the compounds responsible for this activity are concentrated in the DCM and MeOH extracts. Moreover, the different extracts of *S. mombin* were not very cytotoxic at the concentration of 100 µg/mL. This could reassure the population that uses the plant in the treatment of human and animal ailments and in food [5,6].

Unlike *T. leiocarpa* and *S. mombin*, the anthelmintic activity of *M. charantia* and *M. lucida* has been poorly documented. The anthelmintic activity of *M. charantia* has been previously evaluated on *Ascaridia galli*, a digestive parasite of chickens [51]. To our knowledge, this is the first time that the anthelmintic activity of *M. charantia* has been evaluated on *H. contortus*. The MeOH extract of the plant strongly inhibited the migration of *H. contortus* larvae (Figure 5). The results of the phytochemical screening showed that *M. charantia* contains tannins, saponosides, triterpenoids, coumarins, and flavonoids (Table 2) which can justify, in part, this strong anthelmintic activity. Unfortunately, the extracts of *M. charantia* were very cytotoxic except for the H_2_O extract (Figure 7). These results compromise the use of the plant in traditional medicine, but fractionation of the MeOH extract can be undertaken with a view to ensuring that the compounds responsible for the cytotoxicity are different from those responsible for the anthelmintic activity. Similar to *M. charantia*, the MeOH extract of *M. lucida* strongly inhibited larval migration. The anthelmintic activity of *M. lucida* has been little evaluated. The plant is best known for the treatment of malaria, leishmaniasis, and trypanosomiasis [52,53,54]. Several anthraquinones have been identified and isolated as compounds responsible for these biological activities of the plant [52,53,54]. The results of the secondary metabolite quantification show that different extracts of *M. lucida* contained TPC and TFC (Table 5). The MeOH extract was the richest in TPC and TFC which could justify the higher anthelmintic activity of this extract compared to other extracts of the same plant. Unfortunately, the DCM and MeOH extracts were very cytotoxic and compromise the valorization of this plant in the treatment of the digestive parasitosis of small ruminants.

Similar to *M. lucida* and *M. charantia*, the anthelmintic activity of *V. doniana* and *C. bonduc* has been little documented. Indeed, the anthelmintic activity of *V. doniana* extracts has been previously evaluated on *Caenorhabditis elegans* [17]. Similarly, the anthelmintic activity of *C. bonduc* extracts has been evaluated on adult worms of *Pheretima posthuma* (earthworms) and *A. galli* (digestive parasite of chickens) [55]. To our knowledge, this is the first time that the anthelmintic activity of the extracts of both plants has been evaluated on *H. contortus*. Our results show that the H_2_O extract of *V. doniana* was more effective in inhibiting larval migration and is not cytotoxic. This could reassure farmers who use this plant in the treatment of digestive parasitosis of ruminants and even in human food [7]. On the other hand, the extracts of *C. bonduc* were very cytotoxic (except the H_2_O extract). The MeOH and H_2_O extracts of *C. bonduc* were more effective than other extracts of the same plant in inhibiting the migration of *H. contortus* larvae (Table 4).

In summary, our results confirm the known anthelmintic properties of *T. leiocarpa*, *S. mombin*, *Z. zanthoxyloïdes*, and *A. indica* previously described on various nematode and trematode parasites of ruminants on samples from Benin [10,12,14,17]. Furthermore, our results show, for the first time, the anthelmintic activity of *M. charantia*, *M. lucida*, *C. febrifugea*, *A. digitata*, *V. doniana*, and *C. bonduc* on *H. contortus* larvae. The presence of tannins, flavonoids, and triterpenoids in most of the plants studied had been previously reported [27,56,57] and may partly explain the observed anthelmintic activity. The strong cytotoxicity observed in some extracts of *Z. zanthoxyloïdes*, *M. lucida*, *M. charantia*, *C. bonduc*, and *A. indica* corroborates the previous results obtained on various cell lines (breast cell (MCF-7), colon cell (CACO2), liver cell (WRL-68), and prostate cell (PC-3)) [19,20,21,57].

## 5. Conclusions

Our results show that the H_2_O extracts of *V. doniana*, *M. lucida*, *C. bonduc*, *T. leiocarpa*, *A. digitata*, the MeOH extracts of *C. febrifuga*, *T. leiocarpa*, *S. mombin*, *A. digitata,* and the DCM extract of *T. leiocarpa* had better anthelmintic activity and less cytotoxicity. These results provide scientific information that can guide better valorization of the anthelmintic potential of these plants. Similarly, these extracts are potential sources of new anthelmintic molecules. The results of the phytochemical screening showed that the plants studied contain tannins, flavonoids, triterpenoids, and saponosides which may justify, in part, the anthelmintic activity of the different extracts. The MeOH extracts of *M. charantia* and *M. lucida* strongly inhibited the migration of larvae, but their cytotoxicity compromises their use in the treatment of the digestive parasitosis of small ruminants. However, fractionations of these extracts can be undertaken with a view to ensuring that the compounds responsible for their cytotoxicity are different from those responsible for their anthelmintic activities or are degraded in the digestive tract. This may also allow the identification of new anthelmintic molecules to challenge the development of parasite resistance to synthetic anthelmintic molecules.

## Figures and Tables

**Table 1:** Plants studied and their authenticated numbers.

Authenticated Number	Scientific Name	Family
AAC 1504/HNB	*Terminalia leiocarpa* (DC.) Baill.	Combretaceae
AAC 1510/HNB	*Momordica charantia* L.	Cucurbitaceae
AAC 1508/HNB	*Caesalpinia bonduc* (L.) Roxb.	Fabaceae
AAC 1505/HNB	*Adansonia digitata* L.	Malvaceae
AAC 1509/HNB	*Azadirachta indica* A. Juss.	Meliaceae
AAC 1503/HNB	*Crossopteryx febrifuga* (Afzel. ex G. Don) Benth.	Rubiaceae
AAC 1501/HNB	*Zanthoxylum zanthoxyloïdes* (Lam.) Zepern. & Timler	Rutaceae
AAC 1507/HNB	*Morinda lucida* Benth.	Rubiaceae
AAC 1511/HNB	*Vitex doniana* Sweet	Verbenaceae
AAC 1506/HNB	*Spondias mombin* L.	Anacardiaceae

**Table 2:** Phytochemical screening of the selected plants.

Secondary Metabolites	Anacardiaceae	Combretaceae	Cucurbitaceae	Fabaceae	Malvaceae	Meliaceae	Rubiaceae		Rutaceae	Verbenaceae
	*S. mombin*	*T. leiocarpa*	*M. charantia*	*C. bonduc*	*A. digitata*	*A. indica*	*C. febrifuga*	*M. lucida*	*Z. zanthoxyloïdes*	*V. doniana*
Total tannins	+	+	+	+	+	+	+	+	+	+
Catechic tannins	-	-	-	-	-	-	-	-	-	+
Gallic tannins	+	+	+	+	+	+	+	+	+	+
Anthocyanins	-	-	-	-	+	-	+	-	-	-
Leucoanthocyanins	-	-	-	-	+	-	+	-	-	+
Reducing compounds	+	+	+	+	+	+	+	+	+	+
Mucilage	-	-	-	-	+	-	+	-	-	-
Saponosides	-	+	+	-	-	+	+	+	-	+
Cyanogenic derivatives	-	-	-	-	-	-	-	-	-	-
Triterpenoids	+	-	+	+	-	+	-	+	+	+
Steroids	-	+	-	-	-	-	-	-	-	-
Coumarins	+	-	+	-	-	-	-	-	+	-
Quinone derivatives	+	-	-	-	-	-	-	+	+	+
Free anthracene	-	-	-	-	-	-	-	-	-	-
O-Heterosides	-	-	-	-	-	-	-	-	-	-
C-Heterosides	-	-	-	-	-	-	-	-	-	-
Alkaloids	+	-	-	+	-	-	-	-	+	+
Flavonoids	+	+	+	+	-	+	+	+	-	+

+: detected. -: not-detected.

**Table 3:** Yield (% *w*/*w*) of extractions.

	Solvents	Hexane	Dichloromethane	Methanol	Water
Plants					
*Z. zanthoxyloïdes*		1.4	0.6	3.2	6.8
*V. doniana*		3.2	0.9	4.7	8.4
*S. mombin*		1.7	2.5	4.9	8.7
*M. lucida*		4.3	2.1	9.6	13.5
*M. charantia*		0.5	1.4	4.8	14.8
*C. febrifuga*		1.7	3.5	11.8	10.7
*C. bonduc*		5.3	6.6	4.7	9.8
*A. indica*		0.8	8.7	3.6	10.7
*T. leiocarpa*		0.9	16.2	5.6	10.2
*A. digitata*		1.5	7.5	3.7	2.0

**Table 4:** Migration inhibition rate (in %) of infective *Haemonchus contortus* larvae (L3) and viability (in %) of WI38 cells (treated at a concentration of 100 µg/mL) according to the extracts of the plants studied.

Plants	Solvent	Migration Inhibition Rate (%)				Cell Viability (%)
		Concentrations Tested (µg/mL)				
		150	300	600	1200	
*Zanthoxylum zanthoxyloïdes*	HEX	2.3 ± 1.3	3.0 ± 0.3	4.5 ± 1.5	3.0± 2.3	30.3 ± 18.2
	DCM	4.7 ± 1.7	7.0 ± 1.0	17.9 ± 1.0	47.7 ± 3.1	22.7 ± 14.3
	MeOH	14.9 ± 1.0	19.5 ± 1.8	32.2 ± 2.1	29.9 ± 1.3	75.4 ± 2.1
	H_2_O	2.3 ± 1.4	12.6 ± 2.7	12.6 ± 2.7	18.4 ± 2.5	52.1 ± 10.9
*Vitex doniana*	HEX	1.5 ± 1.3	3.8 ± 1.8	8.3 ± 3.5	11.3 ± 2.0	55.2 ± 10.3
	DCM	2.9 ± 1.3	10.8 ± 2.0	13.3 ± 0.9	26.6 ± 1.2	59.2 ± 2.6
	MeOH	2.8 ± 1.8	11.0 ± 2.1	23.2 ± 2.1	25.9 ± 2.2	93.4 ± 11.9
	H_2_O	9.1 ± 2.5	26.6 ± 0.6	27.7 ± 2.2	71.3 ± 2.0	92.9 ± 4.1
*Spondias mombin*	HEX	1.8 ± 1.0	7.7 ± 1.1	8.3 ± 3.7	7.7 ± 1.6	56.6 ± 8.4
	DCM	31.6 ± 1.7	25.1 ± 1.4	36.2 ± 0.6	38.8 ± 1.4	66.3 ± 4.9
	MeOH	9.3 ± 2.5	22.6 ± 2.5	26.6 ± 2.0	33.5 ± 2.9	94.4 ± 10.6
	H_2_O	2.5 ± 2.2	11.4 ± 1.1	13.9 ± 3.3	18.9 ± 2.7	85.4 ± 4.0
*Morinda lucida*	HEX	1.3 ± 1.2	1.9 ± 1.2	3.3 ± 3.0	25.2 ± 2.2	49.3 ± 2.0
	DCM	13.7 ± 2.4	17.1 ± 1.5	20.0 ± 1.0	22.3 ± 3.0	0.6 ± 0.1
	MeOH	18.9 ± 2.8	33.2 ± 1.8	31.1 ± 3.9	43.7 ± 0.8	9.2 ± 8.3
	H_2_O	4.3 ± 1.0	10.1 ± 0.9	20.9 ± 0.6	45.3 ± 2.9	74.8 ± 13.3
*Momordica charantia*	HEX	2.0 ± 1.2	3.36 ± 2.0	16.1 ± 1.2	16.8 ± 2.6	2.9 ± 1.1
	DCM	13.7 ± 2.5	20.0 ± 0.5	28.6 ± 2.1	35.4 ± 0.9	0.7 ± 0.2
	MeOH	16.8 ± 1.7	44.9 ± 1.7	65.9 ± 1.3	63.9 ± 1.9	0.8 ± 0.3
	H_2_O	2.3 ± 0.8	9.4 ± 2.2	15.1 ± 1.3	18.7 ± 2.1	84.9 ± 4.3
*Crossopteryx febrifuga*	HEX	1.9 ± 1.3	2.6 ± 2.0	2.6 ± 2.0	8.6 ± 2.0	64.6 ± 21.7
	DCM	3.9 ± 2.3	21.1 ± 2.2	37.5 ± 2.3	50.8 ± 1.6	44.7 ± 19.1
	MeOH	15.1 ± 0.4	23.9 ± 1.4	33.9 ± 0.6	44.8 ± 1.9	91.8 ± 4.5
	H_2_O	5.3 ± 0.9	4.8 ± 1.8	13.3 ± 2.5	22.3 ± 1.9	90.0 ± 3.8
*Caesalpinia bonduc*	HEX	1.3 ± 1.0	2.9 ± 1.0	5.9 ± 1.6	4.7 ± 3.1	2.9 ± 1.4
	DCM	2.3 ± 2.6	17.9 ± 2.3	18.2 ± 3.3	34.9 ± 2.2	32.9 ± 10.2
	MeOH	10.8 ± 0.6	34.8 ± 1.3	38.6 ± 3.4	47.5 ± 1.4	42.6 ± 11.7
	H_2_O	8.6 ± 1.4	21.5 ± 2.7	28.8 ± 1.4	39.9 ± 2.0	92.7 ± 7.4
*Azadirachta indica*	HEX	7.8 ± 2.2	10.2 ± 0.4	16.3 ± 1.1	21.7 ± 1.1	11.1 ± 1.3
	DCM	12.6 ± 2.0	16.1 ± 1.1	18.4 ± 1.8	27.6 ± 1.9	1.3 ± 0.2
	MeOH	16.1 ± 1.2	17.2 ± 2.0	31.0 ± 0.2	24.1 ± 0.9	80.4 ± 12.3
	H_2_O	10.2 ± 1.4	16.4 ± 0.8	21.8 ± 2.0	21.8 ± 1.3	88.7 ± 6.1
*Terminalia leiocarpa*	HEX	4.2 ± 1.8	6.0 ± 1.6	6.6 ± 0.6	13.3 ± 2.3	68.3 ± 10.0
	DCM	47.8 ± 1.2	52.3 ± 0.5	57.7 ± 1.5	63.5 ± 2.3	91.6 ± 7.6
	MeOH	15.9 ± 1.8	30.6 ± 1.2	52.8 ± 2.9	58.7 ± 2.2	94.4 ± 3.3
	H_2_O	5.9 ± 1.6	26.2 ± 1.6	33.3 ± 3.1	34.1 ± 2.4	94.1 ± 2.7
*Adansonia digitata*	HEX	2.0 ± 1.2	4.0 ± 1.2	6.7 ± 1.2	12.8 ± 1.2	37.2 ± 6.8
	DCM	3.9 ± 2.9	6.4 ± 2.2	14.8 ± 1.4	23.2 ± 2.3	66.4 ± 11.3
	MeOH	19.7 ± 2.1	27.2 ± 2.1	50.0 ± 3.0	60.6 ± 0.7	97.2 ± 5.9
	H_2_O	11.4 ± 2.1	31.6 ± 1.1	54.4 ± 2.3	77.2 ± 1.9	90.8 ± 4.2

HEX: hexane, DCM: dichloromethane, MeOH: methanol, and H_2_O: water. Highlighted lines indicate the most active and less cytotoxic extracts.

**Table 5:** Results of the concentration of phenols, flavonoids, and condensed tannins in the selected extracts.

Identification	TPC (mg GAE/g of Extract)	TFC (mg QE/g of Extract)	CTC (mg CE/g of Extract)
*T. leiocarpa* (HEX)	9.9 ± 1.5	9.6 ± 1.2	0.7 ± 0.1
*T. leiocarpa* (DCM)	268.7 ± 7.9	48.6 ± 1.3	0.6 ± 0.1
*T. leiocarpa* (MeOH)	265.4 ± 6.9	51.3 ± 0.5	1.7 ± 0.5
*T. leiocarpa* (H_2_O)	453.5 ± 2.8	67.9 ± 0.1	6.1 ± 0.3
*C. febrifuga* (HEX)	9.9 ± 0.4	2.4 ± 1.2	0.4 ± 0.0
*C. febrifugea* (DCM)	34.1 ± 2.1	6.39 ± 0.13	0.2 ± 0.0
*C. febrifuga* (MeOH)	98.3 ± 1.5	21.3 ± 0.3	5.2 ± 0.2
*C. febrifuga* (H_2_0)	123.5 ± 6.5	14.4 ± 0.3	8.5 ± 0.7
*M. lucida* (HEX)	14.0 ± 1.7	8.6 ± 3.4	0.5 ± 0.1
*M. lucida* (DCM)	22.4 ± 1.9	5.4 ± 0.9	0.1 ± 0.0
*M. lucida* (MeOH)	42.5 ± 1.4	10.5 ± 1.5	0
*M. lucida* (H_2_O)	30.7 ± 0.9	9.9 ± 1.5	0

TPC: total phenolic content, TFC: total flavonoid content, CTC: condensed tannin content, GAE: gallic acid equivalents, QE: quercetin equivalents, CE: catechin equivalents, HEX: hexane, DCM: dichloromethane, MeOH: methanol, and H_2_O: water.

## Data Availability

All data generated or analyzed during this study are included in this article.
